# Analysis with LC-OCT imaging in split face with CO_2_ laser and 1540 nm. A case report

**DOI:** 10.1093/omcr/omaf296

**Published:** 2026-02-18

**Authors:** Francesco Moro, Irene Fusco, Francesca Madeddu, Luca Ambrosio, Camilla Chello, Giovanni Di Lella, Tiziano Zingoni, Laura Colonna

**Affiliations:** IDI-IRCCS, Dermatological Research Hospital, Via dei Monti di Creta, 104, Rome 00167, Italy; Department of Clinical Research and Practice, El.En. Group, Via Baldanzese 17, Calenzano 50041, Italy; Department of Clinical Research and Practice, El.En. Group, Via Baldanzese 17, Calenzano 50041, Italy; IDI-IRCCS, Dermatological Research Hospital, Via dei Monti di Creta, 104, Rome 00167, Italy; Dermatology Unit, Department of Clinical Internal Anesthesiologic Cardiovascular Sciences, “Sapienza” University of Rome, Viale Regina Elena 334, Rome 00185, Italy; IDI-IRCCS, Dermatological Research Hospital, Via dei Monti di Creta, 104, Rome 00167, Italy; Dermatology Unit, Department of Clinical Internal Anesthesiologic Cardiovascular Sciences, “Sapienza” University of Rome, Viale Regina Elena 334, Rome 00185, Italy; IDI-IRCCS, Dermatological Research Hospital, Via dei Monti di Creta, 104, Rome 00167, Italy; Department of Clinical Research and Practice, El.En. Group, Via Baldanzese 17, Calenzano 50041, Italy; IDI-IRCCS, Dermatological Research Hospital, Via dei Monti di Creta, 104, Rome 00167, Italy

**Keywords:** simultaneous laser emission, split-face, confocal optical coherence tomography

## Abstract

**Background/Objective:**

The aims of this case report study was to investigate the effect of both ablative and non-ablative laser sources in the restoration of the epidermal profile with split-face confocal optical coherence tomography (LC-OCT) evaluation.

**Methods:**

A patient was treated with a standard laser treatment that use simultaneously both ablative and non-ablative sources in split face mode; one side with CO_2_ alone and the other with the combined use of CO_2_ and a 1540 nm emission. LC-OCT imaging was carried out before the procedure (T0), at 24 hours (T1), and at 7 days (T7).

**Results:**

In the CO_2_ + 1540 nm modality, the general appearance was comparable to the CO₂-only side. At T7, the CO_2_-only side demonstrated substantial clinical recovery, while the combined CO_2_ + 1540 nm side shows faster re-epithelialization. This split-face LC-OCT evaluation highlights that both treatment modalities induce comparable early inflammatory changes.

## Introduction

The use of fractional CO_2_ lasers for rejuvenation and scar revision has already abundantly demonstrated its effectiveness in terms of healing quality and patient compliance. This technique makes it possible to generate areas of micro thermal damage (MTZ) surrounded by non-irradiated tissue and speed up healing and the containment of side effects. In fact, over two days, fibrin caps are formed that protect the vaporized area from foreign agents [1].

The advantage of infrared ablative laser systems is also to be found in the additional non-ablative controlled thermal effect that develops around the micro-ablation zones (MAZ) that define the micro thermal zone (MTZ), providing an additional stimulation tool for a contraction, a denaturation of the collagen fibers and a reversible thermal effect through the cytokine cascade [2].

Although recent technologies allow a refined flexibility of the system, the nature of the wavelength at 10600 nm imposes a certain proportionality and dependence between the ablation zone and the thermal zone that constrain the reciprocal increase of the two areas as the energy delivered increases [3].

Both ablative and non-ablative wavelengths such as radiofrequency or infrared in the range 1540–1570 nm have therefore been used in combination to split this dual effect and make the systems even more flexible to perform better procedure [4].

This combination has been able to make the morphologies of the two areas of competence of the ablative and non-ablative laser almost independent, allowing to stimulate even depths of 3–4 mm in a thermal way without the need to get there with the ablative method [5].

This has allowed an increase in efficacy performance, which has been able to balance the two effects based on the type of treatment to be performed, customizing the approach to the individual imperfection and at the same time has also introduced a significant acceleration of the healing of the lesion attributable to MTZ by increasing cell turnover, thus allowing greater safety in the treatment by limiting the exposure of healing [6].

## Case report

In this case report, we treated a patient with a standard laser treatment that use simultaneously both ablative and non-ablative sources (DuoGlide, Deka Mela, Florence, Italy) in split face mode; one side with the use of CO_2_ alone (Power 15 W, Spacing 500 um, D-Pulse, Dwell time 1000 us) and the other with the combined use of CO_2_, with the same parameters) and a 1540 nm emission (Power 6 W, Dwell Time 5 ms). For the evaluation of the healing, line-field confocal optical coherence tomography (LC-OCT) imaging (DeepLive™, DAMAE Medical, France) was carried out before the procedure (T0), at 24 hours (T1), and at 7 days (T7).

At baseline (T0), LC-OCT revealed a well-defined dermo-epidermal junction and a fibrillar dermal collagen pattern with slightly hyper-reflective features.

At T1, both treated areas demonstrated disruption of the epidermal and dermal silhouette. In both treatment modes, peri-ablative columns of the CO_2_ laser showed similar inflammatory hypercellularity, visualized as multiple white dots surrounding the ablation craters. In the combined CO_2_ + 1540 nm modality, the general appearance was comparable to the CO_2_-only side; however, in the inter-crater space, a more pronounced reactive hypercellularity was observed, consistent with histological findings reported in previous studies.

At T7, the CO_2_-only side demonstrated substantial clinical recovery. In LC-OCT vertical sections, the ablation columns were less evident, and the overall epidermal-dermal silhouette was nearly reconstituted. Dermal collagen appeared more reflective, indicating early remodeling, although a mild persistence of inflammatory cells was still visible.

**Figure 1:**
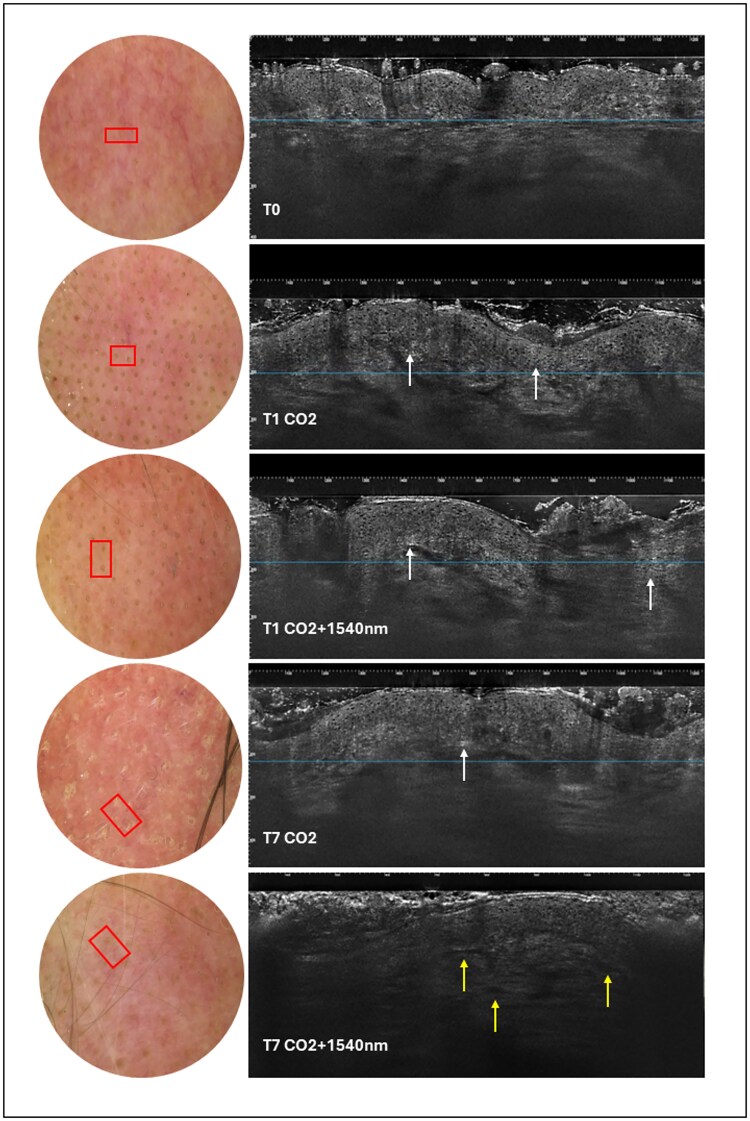
LC-OCT split-face evaluation. At T1 both CO_2_ and CO_2_ + 1540 nm sides show peri-ablative inflammatory hypercellularity (white arrows). At T7, faster epidermal repair is visible with CO_2_ + 1540 nm with greater dermal vascular expression (yellow arrows), while CO_2_ alone shows near-complete dermo-epidermal reconstitution.

In contrast, the combined CO_2_ + 1540 nm side shows faster re-epithelialization, resulting in a more complete restoration of the epidermal profile. All results are shown in Fig. 1. Nevertheless, persistent inflammatory activity at the dermal level was observed, with greater vascular expression compared to the CO_2_-only side. This suggests a more substantial and more prolonged reactive component induced by the dual-wavelength protocol, potentially translating into enhanced long-term collagen remodeling.

## Discussion

This split-face LC-OCT evaluation highlights that both treatment modalities (CO_2_ alone and CO_2_ combined with 1540 nm) induce comparable early inflammatory changes. However, the combined approach appears to promote faster epidermal repair while maintaining a more sustained dermal inflammatory and vascular response. These findings agree with previous histological evidence suggesting that the addition of a non-ablative infrared wavelength enhances dermal remodeling potential.

## Data Availability

Data available from the corresponding author (IF) upon reasonable request.
